# Sound Guides Object Size Choices in African Penguins Through Pitch–Size Association

**DOI:** 10.1111/nyas.70313

**Published:** 2026-06-11

**Authors:** Francesca Terranova, Francesca D'Orazio, Cristina Pilenga, Luigi Baciadonna, David Reby, Livio Favaro

**Affiliations:** ^1^ Department of Life Sciences and Systems Biology University of Turin Turin Italy; ^2^ Department of Human Neurosciences, Faculty of Medicine and Dentistry Sapienza University of Rome Rome Italy; ^3^ Zoomarine Openature Rome Torvaianica Italy; ^4^ ENES Bioacoustics Research Laboratory, Lyon Neuroscience Research Centre (CRNL) Jean Monnet University Saint‐Etienne France; ^5^ Institut universitaire de France Paris France

**Keywords:** bioacoustics, bird cognition, multimodal association, *Spheniscus demersus*

## Abstract

The brain's ability to associate information from different sensory modalities, known as cross‐modal integration, is crucial for forming a coherent perception of objects and events. When such associations follow systematic and predictable patterns, they are referred to as cross‐modal correspondences. Humans, for example, tend to associate large objects with low‐pitched sounds and small objects with high‐pitched sounds, a phenomenon known as pitch–size cross‐modal correspondence. This phenomenon has been documented in several vertebrate species, yet direct experimental evidence in avian species remains comparatively limited. Here, we tested African penguins using a preferential choice paradigm in which individuals chose between two cubes of different sizes after hearing either a high‐ or low‐pitched sound. Following passive exposure to congruent pitch–size pairings, penguins chose larger objects following low‐pitched sounds and smaller objects following high‐pitched sounds, indicating that auditory pitch guided their visual size choices. Given that individuals were pre‐exposed to a limited set of pitch–size pairings, this effect may reflect either a preexisting bias consistent with cross‐modal correspondences or the generalization of a learned association. Nevertheless, these findings demonstrate that penguins can integrate auditory and visual information to guide their behavior and generalize a systematic pitch–size relationship across sensory dimensions.

## Graphical abstract

African penguins use auditory pitch to guide visual size choices. When hearing high‐pitched sounds, they preferentially approach and gaze at smaller objects, and when hearing low‐pitched sounds, they preferentially approach and gaze at larger ones. This pitch–size association may support coherent expectations about the physical characteristics of signalers in a species whose vocal signals encode body size across life stages.

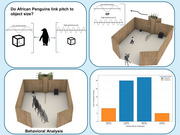

## 1 Introduction

Animals constantly receive information from multiple sensory channels simultaneously. The ability to integrate this information is essential for constructing a unified and coherent perception of the surrounding environment [1, 2, 3]. This cross‐modal integration reduces perceptual uncertainty and supports adaptive decision‐making in complex natural settings [4, 5]. When specific features from different sensory modalities are systematically associated in a predictable manner, these links are termed cross‐modal correspondences [6]. These correspondences typically occur between polarized dimensions, where extreme values on one continuum (e.g., high or low) are matched with corresponding extremes on another.

In humans, one of the most robust and well‐studied examples is the pitch–size correspondence: people reliably associate low‐pitched sounds with large objects and high‐pitched sounds with small objects [7, 8, 9, 10]. This association is part of a broader network of cross‐modal correspondences that includes associations between pitch and visual brightness, spatial height, and luminance, as well as links between other sensory qualities such as texture and color, or shape and taste [11, 12, 13, 14, 15, 16, 17, 18, 19]. These correspondences influence a wide range of perceptual and cognitive processes, from speeded classification tasks to everyday object recognition and aesthetic judgments.

Importantly, cross‐modal correspondences are not restricted to humans. Similar patterns have been observed across a growing number of vertebrate species. Rhesus monkeys associate rising frequency sounds with looming visual stimuli [20], while domestic chicks demonstrate spontaneous associations between luminance and spatial position [21], visual and tactile cues [22], and sound–shape correspondences analogous to the human Bouba–Kiki effect [23]. Reptiles also exhibit cross‐modal abilities: Hermann's tortoises, for instance, show pitch–luminance [24] and pitch–size associations [4]. Pitch–size correspondences specifically have been experimentally demonstrated in chimpanzees [25], domestic dogs [26], and tortoises. In birds, indirect evidence suggests that vocal pitch is used to assess or signal body size: willow warbler males appear to use song pitch to assess rival body size [27], and purple‐crowned fairy‐wren males produce advertising songs whose low‐frequency components vary with body size, consistent with body‐size signaling [28]. However, direct experimental evidence for pitch–size associations in birds from controlled choice paradigms remains comparatively limited.

A key question concerns how these correspondences arise. From a theoretical perspective, they may reflect innate perceptual biases or emerge through multisensory statistical learning—the internalization of statistical regularities present in the natural environment. Through repeated exposure to consistent sensory relationships, individuals build expectations about how cues covary, such that responses extend to novel situations, thereby reducing uncertainty. Over time, these learned associations might become internalized as perceptual biases, as suggested by Bayesian coupling‐prior frameworks [29], which posit that the brain encodes environmental co‐occurrence statistics to bias multisensory perception. In the physical world, these statistical regularities are often grounded in biomechanical and environmental constraints: larger objects tend to produce lower‐frequency sounds, and larger animals typically vocalize at lower fundamental frequencies due to larger vibrating tissues (e.g., vocal folds or syringeal membranes) and longer vocal tracts with lower resonant frequencies [30, 31]. Whether these regularities are internalized through individual development or reflect evolved perceptual biases shaped over evolutionary time remains an open question.

African penguins (*Spheniscus demersus*) provide a particularly valuable model for investigating the evolutionary origins of such mechanisms. As members of the order Sphenisciformes, they diverged from other avian lineages approximately 65 million years ago [32], offering a key comparative perspective on multisensory processing in an early‐diverging bird group. Within this species, acoustic cues to body size are biologically relevant. The fundamental frequency of begging calls in chicks is negatively correlated with body mass and age [33]. In adults of African penguins’ closely related species, Humboldt penguins (*Spheniscus humboldti*) and Magellanic penguins (*Spheniscus magellanicus*), both vocalization duration and fundamental frequency reliably encode body size and mass [34]. Furthermore, African penguins are capable of perceiving variations in fundamental frequency and formant frequencies within species‐specific vocalizations [35] and successfully integrate auditory and visual information to recognize individual conspecifics [36].

Despite growing evidence that African penguins rely on acoustic parameters to infer information about signalers and integrate these cues cross‐modally, it remains unknown whether auditory pitch systematically shapes visual size selection. Here, we investigated whether auditory pitch influences visual size selection in a controlled two‐choice experimental setting. We predicted that if African penguins possess a pitch–size association, they should preferentially approach larger objects when hearing low‐pitched sounds and smaller objects when hearing high‐pitched sounds.

## 2 Methods

African penguins were housed at Zoomarine (Italy). The colony comprised 21 African penguins, ranging from 6 months to 20 years old (Table S1). Each penguin was uniquely identifiable by a colored plastic ring attached to one wing, and the distinctive dot pattern on their ventral side further facilitated individual recognition [37]. The colony was housed in an outdoor exhibit of 70 m^2^, with sand and pebbles and a 228‐m^3^ saltwater pool. Zookeepers hand‐fed the penguins three times daily.

### 2.1 Audio and Visual Stimuli

We prepared controlled audio–visual stimuli for two experimental phases: an exposure phase and a subsequent testing phase.

For the exposure phase, five black cardboard cubes of different sizes were constructed (Figure S1), following evidence that birds can discriminate among cubic objects [38]. Black was chosen to maximize visual salience against the background [39]. Each cube had one removable side allowing the inside placement of a Bose SoundLink Mini II speaker connected to a Redmi Note 10 Lite smartphone. Object size was manipulated by varying cube volume. Starting from a medium cube (T0; 21 cm per side), four additional cubes were created by increasing or decreasing the volume by 5% or 10%, considering that birds encode size logarithmically [40]. Acoustic stimuli were pure tones designed to fall within the auditory sensitivity range reported for African penguins [41] and to reflect pitch contrasts comparable to those used in studies of pitch–size correspondences in other vertebrates [26]. Pure tones were generated in Praat v. 6.3.09 [42] with the following features: 2 s duration, 48 kHz sampling frequency, 0.2 Pa amplitude, and 0.1 s fade‐in/out. For the medium cube (T0), we used a 430‐Hz pure tone; the remaining tones were created by increasing or decreasing frequency by 5% and 10% (Figure S1). We employed pure tones to isolate the fundamental frequency as the relevant auditory cue and to avoid introducing other acoustic features (e.g., amplitude modulation or harmonic structure).

**FIGURE 1:**
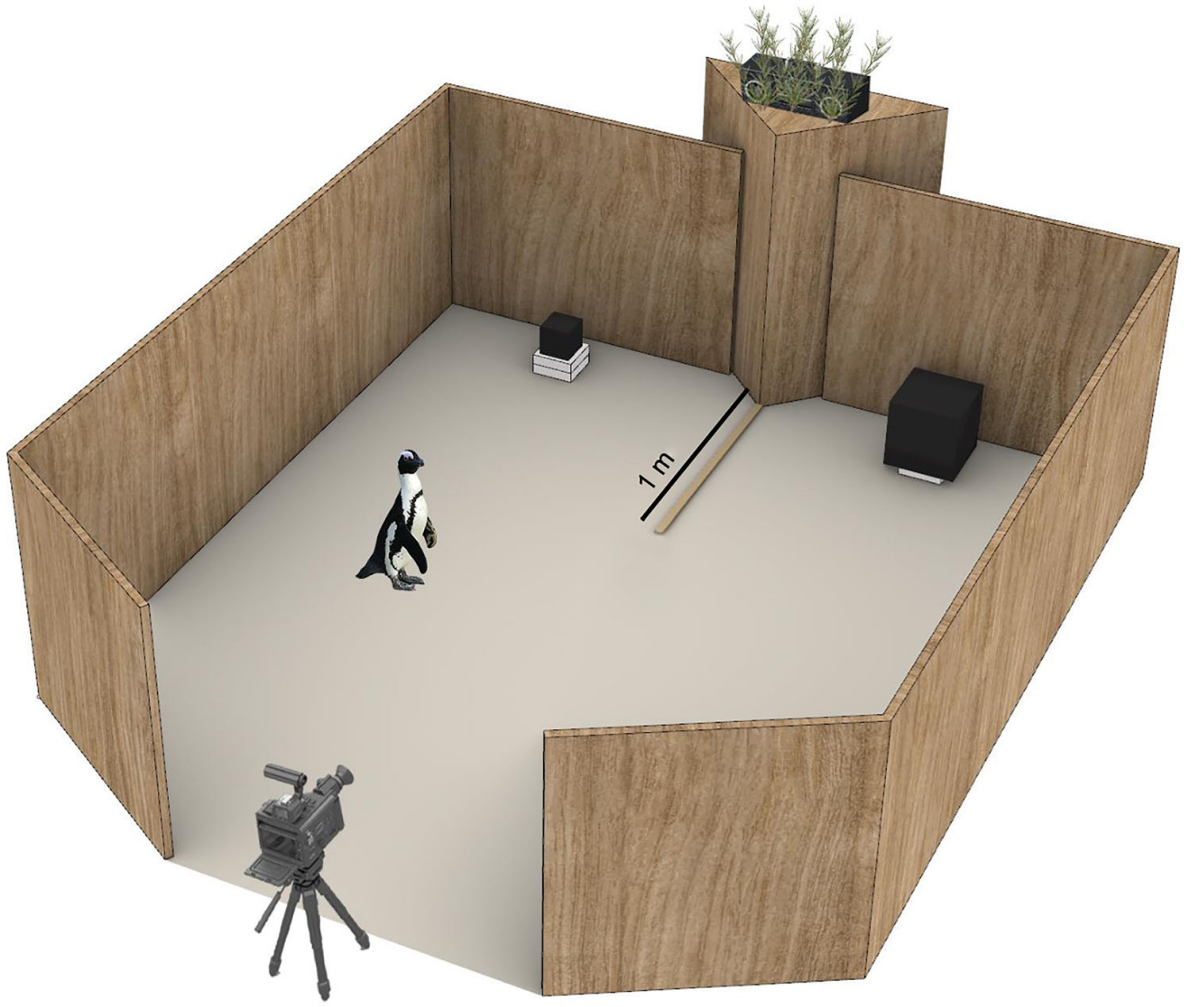
Setup of the arena used in the testing phase. The cubes of different sizes were placed at 170 cm from each other. The penguins were exposed to acoustic stimuli from a hidden speaker positioned elevated between the stimuli. See the Supporting Information (Figure S2) for more details.

For testing, we used two new cube sizes and two novel pitches that were not presented during exposure. The cubes were a large one (28 cm per side) and a small one (13 cm per side; Figure 1), each presented with either a low‐pitch (150 Hz) or high‐pitch (900 Hz) tone.

All sounds were played at a sound pressure level of 65–70 dB, measured at the start of each session using a Monacor SM‐2 sound level meter. We used the C‐weighted scale, which emphasizes low‐frequency sounds, making it suitable for assessing the acoustic characteristics relevant to the study. Measurements were conducted at a height of 70 cm and a distance of 1 m from the speaker, mimicking the position of the penguins’ head during the tests.

### 2.2 Exposure Phase

The exposure phase was designed to ensure that behavioral responses during testing reflected attention to the audiovisual stimuli rather than first‐time novelty reactions, which are common in neophobic, nondomesticated birds such as penguins [43, 44]. This phase comprised 60 short sessions distributed across 30 nonconsecutive days and was conducted during regular feeding times; the last 24 sessions were held inside the wooden‐panel arena later used for testing (Figure 1) to minimize its novelty effects. Importantly, this exposure phase cannot be construed as a training procedure, as penguins were not required to make choices, and all penguins were fed routinely regardless of whether or how they interacted with the stimuli.

During each session, while a keeper hand‐fed the penguins, a single cube was placed in front of the group and paired with a pure tone. Cube size and tone frequency varied slightly across days and covaried in a congruent manner, with larger cubes paired with lower frequencies and smaller cubes with higher frequencies. Exposure occurred in a group setting, penguins were free to approach the stimuli without being separated from their social context, and food delivery followed routine feeding regardless of the penguins’ behavior (Video S1). Individuals that oriented toward or pecked the cube at least once following sound playback were noted and classified as attentive to the stimuli and were subsequently included in the testing phase.

### 2.3 Testing Phase

Testing began immediately after the exposure phase and lasted for 8 days. Of the initial 21 penguins, 19 were selected for the testing phase, with two individuals excluded: one juvenile, which was excluded due to being too young, and one adult, which was absent from the exposure phase because it was incubating the eggs. Each penguin completed four trials in total (no more than one trial per day): two congruent and two incongruent trials, with the target side randomized across trials (Table 1). The order of the four trial types was fully randomized.

**TABLE 1:** Audio–visual stimuli combinations. Each penguin was presented with four unique testing conditions—two congruent and two incongruent—created by a combination of two sounds (high pitch = 900 Hz; low pitch = 150 Hz) with two cubes (small size = 13 cm per side; large size = 28 cm per side).

Pitch	Object size	Object position	Condition type
High	Small	Right	Congruent
		Left	
	Large	Right	Incongruent
		Left	
Low	Small	Right	Incongruent
		Left	
	Large	Right	Congruent
		Left	

Trials took place in a temporary arena built from wooden panels and plywood (Figure 1). The large and small cubes were placed on the ground ∼170 cm apart and at matched height. A speaker was positioned centrally between them, elevated and visually concealed by vegetation. Trials were conducted in the outdoor area of the colony enclosure, minimizing the possibility of echo and reverberation effects. Penguins were guided into the arena from an adjacent holding area by keepers. Once a penguin entered, the exit panel was closed, and the experimenter left the arena to minimize interference. The penguin was given ∼1 min to settle, during which he could move freely within the arena, then the tone for that trial (high or low) was played. From audio onset, the bird's behavior was video recorded for 1 min using a Sony Handycam mounted ∼30 cm above ground and oriented toward the arena entrance. A single audio stimulus was presented per trial. After the trial, the bird was released back into the main nesting area. Example trials are shown in the Supporting Information (Videos S2). We presented both cubes simultaneously because pitch–size association in other vertebrates appears to be relative rather than absolute [26, 45]. Thus, the task required the penguin to orient, look, or approach in the presence of two alternative size options under a single pitch context.

### 2.4 Behavioral Analyses

**FIGURE 2:**
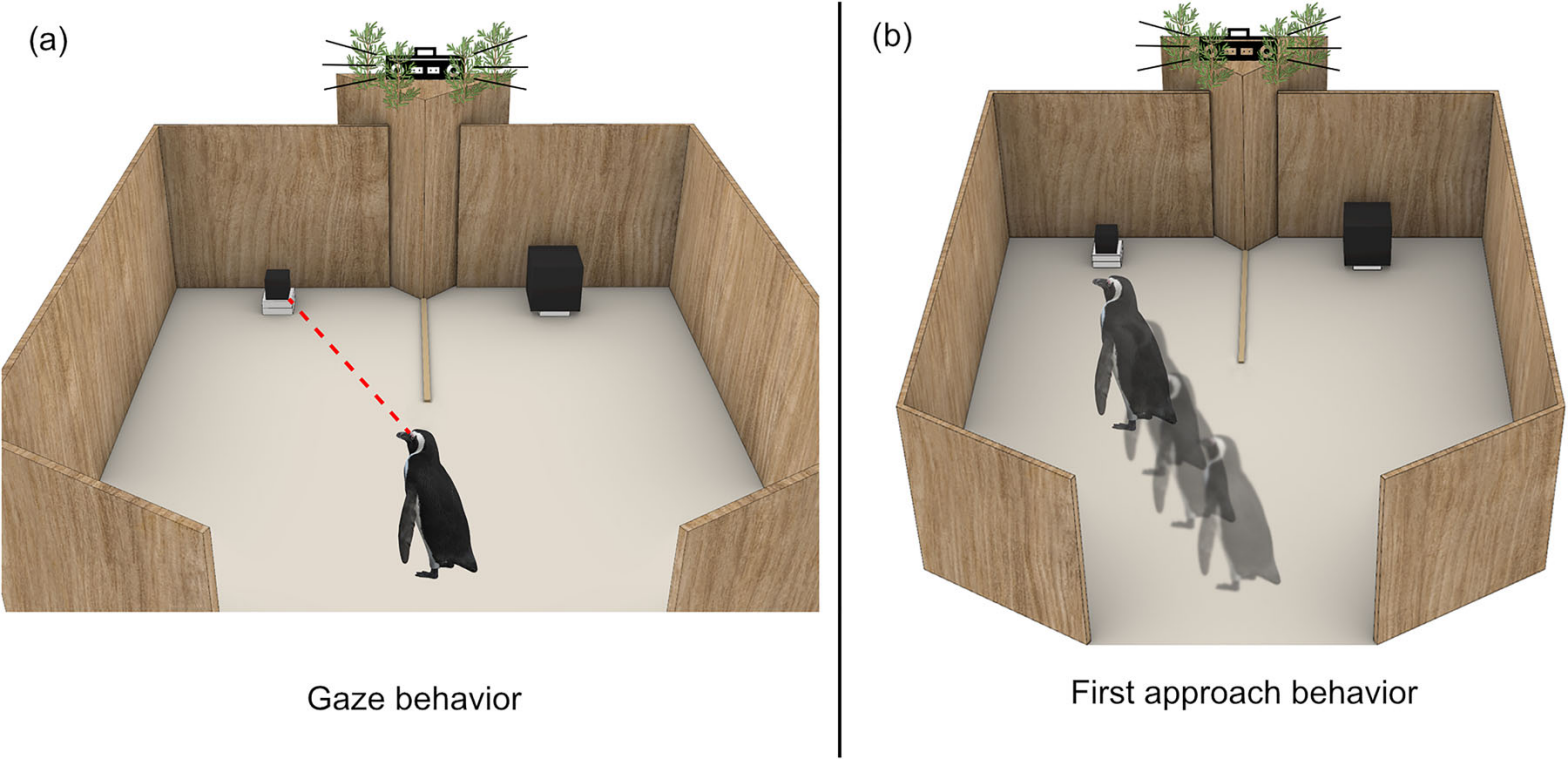
Schematic representation of the two coded behaviors: (a) gaze behavior, showing the penguin's beak orientation toward an object, and (b) first approach behavior, illustrating the penguin's initial movement toward the small object.

Behavioral variables were coded using BORIS v.8.21.8 [46]. Video recordings were analyzed at 0.5x speed with the audio track enabled and the spectrogram was used to precisely identify stimulus onset. Coding began at the onset of the auditory stimulus for each trial, and behaviors were scored according to predefined, objective operational criteria as follows:

- *Gaze duration*: in seconds, how long each penguin observed each cube after tone onset. Gaze direction was assessed based on the orientation of the penguin's beak and scored only if the penguin clearly directed its gaze at one of the two cubes, as African penguins use forward‐facing binocular vision when focusing on objects [37, 47]. Instances in which the penguin looked toward the center of the arena or toward the exit (away from the cubes) were not scored. Gaze duration was measured as a continuous variable, for example, “Penguin looked at the large object on the left for 4.5 s” or “Penguin did not gaze (0 s)” (Figure 2a). Additionally, to measure gaze preference, we calculated the preferential gaze index adapted from Albuquerque et al. [48]. The index is calculated as (𝐶 − 𝐼)/𝑇, where *C* is the total time the penguin gazed at the congruent stimulus, *I* is the total time spent gazing at the incongruent stimulus, and *T* is the total gaze time (congruent gaze + incongruent gaze). Hereafter, “congruent” refers to audio–visual pairs that align with cross‐modal association (e.g., a large object with low pitch, a small object with high pitch), while “incongruent” refers to the reverse combinations.
- *First approach*: we recorded which cube, if any, the penguin first approached after tone onset. An approach was defined as the bird crossing into the half of the arena containing that cube (delineated by the central wooden divider). Movements to the center of the arena or near the exit were not scored. Only the first approach was recorded. This behavior was coded as a binary variable: either the penguin approached a specific object (e.g., a small object positioned on the right after a high‐pitched sound) or did not approach (Figure 2b). Based on this behavior, we considered two aspects: object size choice indicating whether the penguin approached the large or small cube and selected stimuli combination, which refers to the pairing of the chosen cube with the specific audio condition in each trial (e.g., a large cube paired with a low‐pitched sound).

To ensure reliability, 10% of the videos were scored by a second observer. Blind scoring was not feasible, as identifying behavioral onset required access to the audio track, which made the pitch condition visible to scorers. Interrater reliability was strong, with a kappa coefficient of 1 for the first approach, indicating perfect agreement. For the preferential gaze index, the Pearson correlation between raters was also high, with a correlation coefficient of 0.84.

### 2.5 Statistical Analysis

Statistical analyses were conducted in RStudio (v. 4.1.3). We analyzed the first approach behavior to test whether object size choice varied with the pitch played. We used a chi‐square test with Yates’ correction to evaluate whether penguins’ choices of cube size (large vs. small) were associated with the audio condition (high vs. low). We then fit a generalized linear mixed model (GLMM; package *glmmTMB* [49]) to explore how the audio condition influenced object size choice. Object size (large or small) was treated as the binary response variable, with audio condition (high or low) and large object position (right or left) as fixed factors. The inclusion of large object position aimed to evaluate whether penguins exhibited an attentional bias toward the large object, potentially due to its visual salience [50]. Individual ID and session were included as random effects. We verified statistical assumptions for using *glmmTMB*, confirming no collinearity among fixed factors. The significance of the full model was evaluated using a chi‐square test [51], and a *drop1* analysis was performed to identify the fixed factors influencing the response [52].

To evaluate the penguins’ preferential gaze behavior toward objects under different audio conditions, we first aggregated individual subjects’ preferential gaze behavior data (Table S2). We then conducted the one‐sample *t*‐test on the aggregated scores to determine whether the mean preferential gaze index (ranging from −1 to 1) was significantly different from zero (absence of preference). This test assessed if penguins displayed an overall preference for congruent audio–visual pairings (e.g., large objects with low‐pitched sound). Additionally, we calculated each subject's mean preferential gaze behavior separately for the low and high audio conditions (Table S3). This aggregation allowed us to obtain a single average preferential gaze score per audio condition for each penguin. Subsequently, one‐sample *t*‐tests were conducted separately for each audio condition (high‐pitched and low‐pitched) to investigate the preference for a specific audio condition. All assumptions for the *t*‐tests were verified prior to analysis. Normality was assessed using the Shapiro–Wilk test.

## 3 Results

**FIGURE 3:**
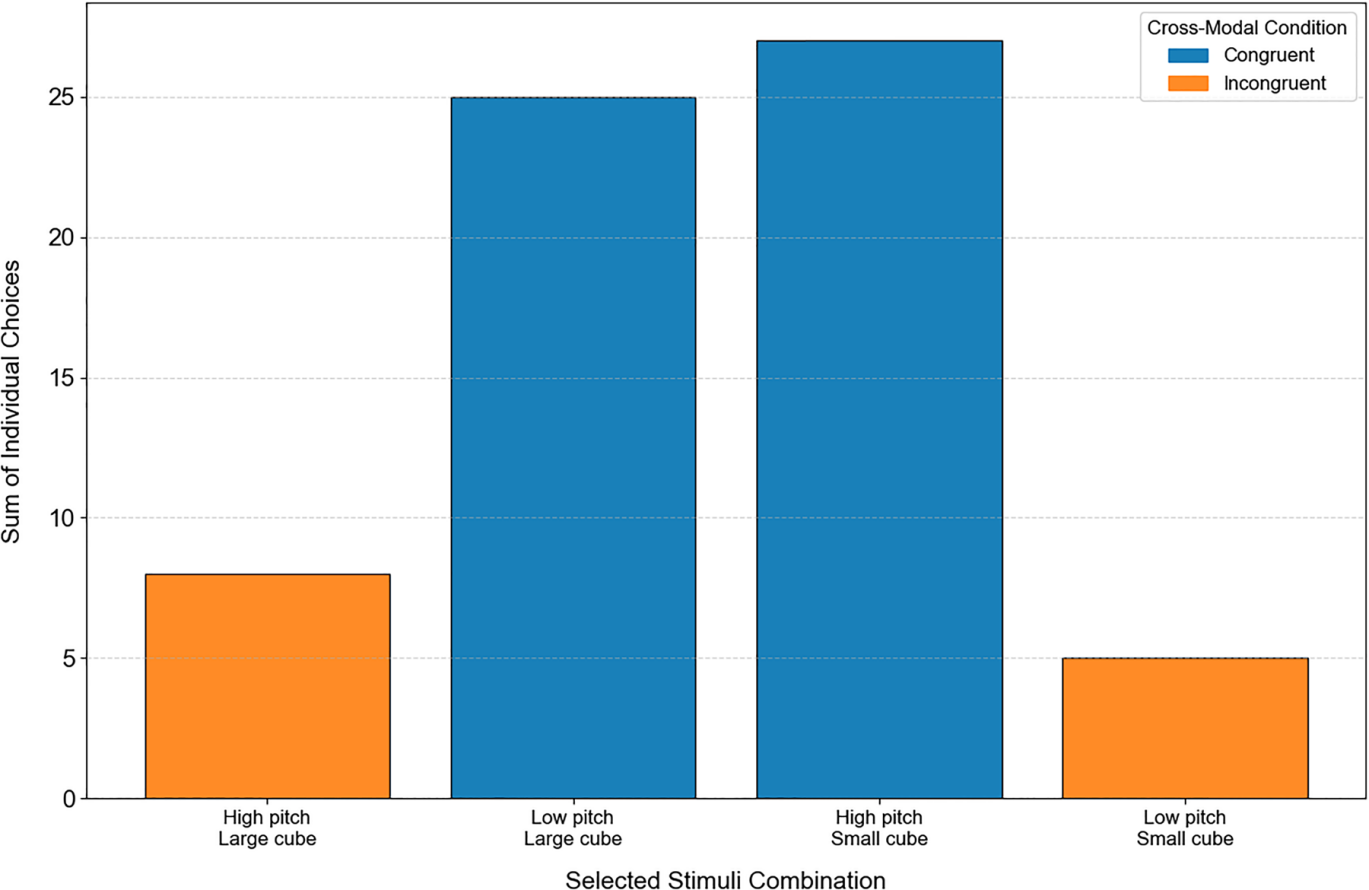
Sum of individual choices of African penguins for different stimuli combinations under congruent and incongruent audio–visual conditions.

We analyzed penguins’ first‐approach behavior across 72 trials to assess whether their responses varied with the pitch of the auditory cue. Penguins approached one of the two stimuli in 65 trials, while the remaining trials showed no approach. Among the 65 approach responses, penguins directed their first approach toward the size–pitch combination consistent with the congruent pitch–size pairings (large object with low pitch, or small object with high pitch) in 80% of cases (Figure 3).

The chi‐square test confirmed a statistically significant association between auditory pitch and object size choice (*χ* ^2^ = 21.28, *p* < 0.001), with high‐pitched sounds biasing choices toward smaller objects and low‐pitched sounds toward larger objects.

Results from the GLMM analysis corroborated this pattern: approach direction was significantly influenced by the audio condition (GLMM full vs. null: *χ* ^2^ = 15.29, df = 2, *p* < 0.001). The drop1 function confirmed that audio condition was a significant predictor of object size choice while the large object position was not significant. The summary of the model is presented in Table 2.

**TABLE 2:** Summary of the generalized linear mixed model examining the influence of *audio condition* and *large object position* on *object size choice*.

	Estimate	SE	*Z*‐value	*p*‐value
(Intercept)	1.32	0.78	1.69	0.09
Audio condition (low)	−4.33	1.45	−2.99	0.003^**^
Large object position	1.18	0.82	1.44	0.15

*Note*: ** indicate statistical significance at p < 0.01.

Penguins approached the object whose size was consistent with the pitch–size association. Specifically, the estimated probability of approaching the smaller object was significantly higher with the high‐pitched auditory cue (0.87; 95% CI: 0.57–0.97) compared to the low‐pitched auditory cue (0.08; 95% CI: 0.01–0.37). Conversely, the estimated probability of choosing the larger object was higher with the low‐pitched cue (0.91; 95% CI: 0.63–0.99) than with the high‐pitched cue (0.13; 95% CI: 0.03–0.43; Figure S3).

**FIGURE 4:**
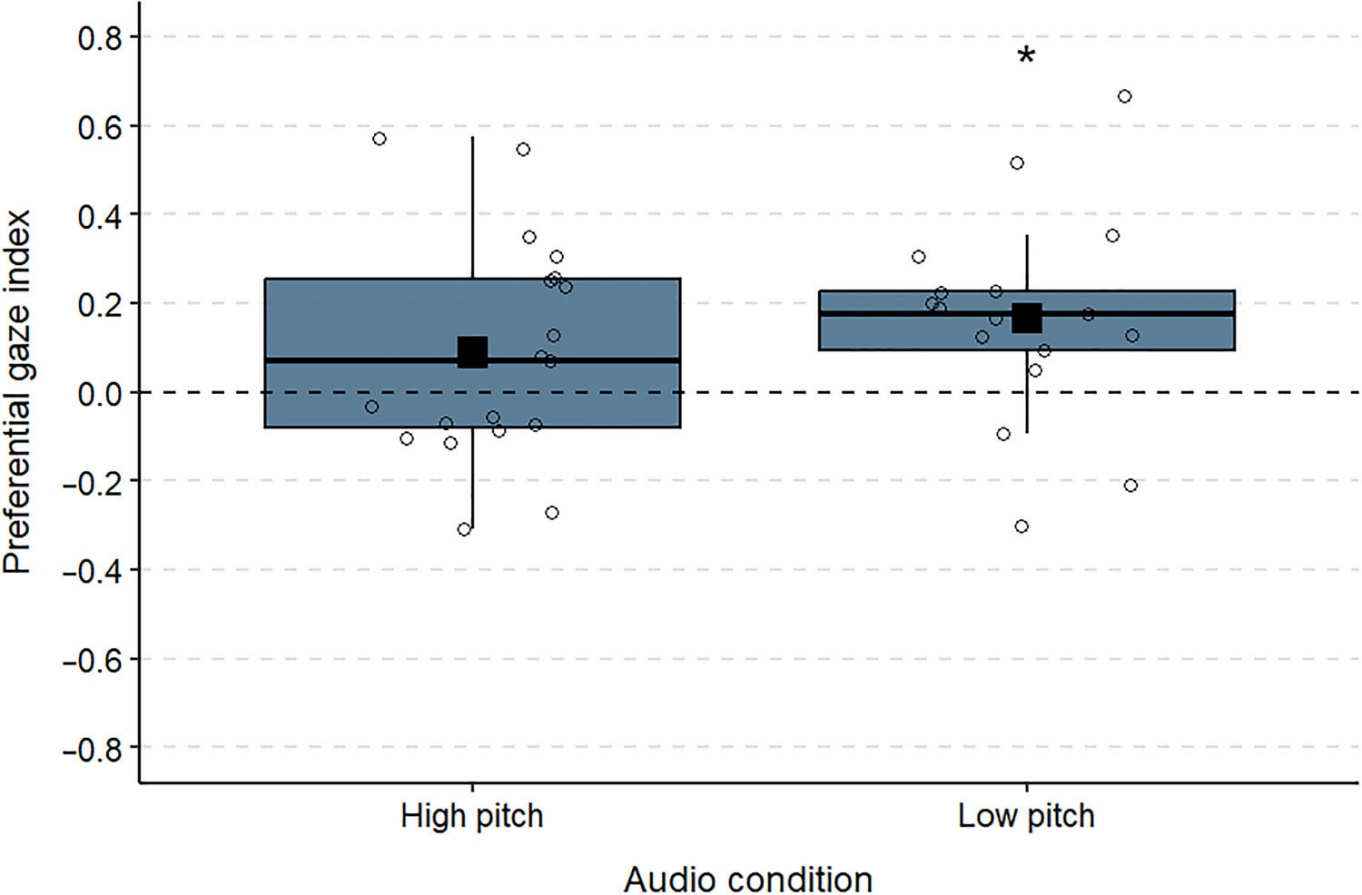
Preferential gaze index of penguins across the two audio conditions. Higher values indicate a stronger preference for congruent audio–visual stimuli combinations. The box plots display the median (horizontal line) and interquartile range (box height) for each condition, with individual points representing penguin averages and black squares indicating the mean. The dashed line at zero represents a neutral gaze index. The asterisk (*) indicates a significant difference from zero (one‐sample *t*‐test, *p* < 0.05).

The analysis of gaze behavior showed a similar pattern. The mean preferential gaze index across individuals was *M* = 0.12 ± 0.19 SD (95% CI: 0.03–0.22, *n* = 19). This value was significantly greater than zero (one‐sample *t*‐test: *t* = 2.74, df = 18, *p* = 0.0067), indicating a general gazing preference for congruent audio–visual pairings (e.g., larger objects with low‐pitched sounds and smaller objects with high‐pitched sounds) (Figure 4). The one‐sample *t*‐test for low‐pitched sounds indicated a significant positive gaze preference, with a mean preferential gaze index significantly greater than 0 (*t* = 2.91, df = 16, *p* = 0.0051). In contrast, the one‐sample *t*‐test for high‐pitched sounds did not reach statistical significance, suggesting no strong preference in this condition (*t* = 1.55, df = 18, *p* = 0.069). This indicates that African penguins exhibit a significant gaze preference for low‐pitched sounds, whereas high‐pitched sounds do not elicit a similar response.

## 4 Discussion

In this study, we investigated whether African penguins systematically associate auditory pitch with visual object size. Following passive exposure to congruent pitch–size pairings, the penguins were tested with novel combinations of object size and sound pitch. During testing, individuals preferentially approached and gazed at larger objects when hearing low‐pitched sounds and smaller objects when hearing high‐pitched sounds. This pattern demonstrates that African penguins successfully generalized a pitch–size relationship across sensory modalities and applied it to previously unseen stimuli.

Such dimensional relationships between auditory pitch and visual size are widely documented within the framework of cross‐modal correspondences [6]. Our findings also highlight that pitch–size congruent behavior extended to novel stimuli following passive, unsupervised exposure, whereby features in one sensory modality are systematically linked to features in another. Because the exposure phase introduced pitch–size covariation, the present design does not allow us to determine whether the observed pattern reflects a preexisting perceptual correspondence or the acquisition of a learned association during the experiment. Regardless of its developmental origin, pitch–size congruent behavior extended to novel stimuli, demonstrating sophisticated multisensory processing in this species.

Our results extend previous demonstrations that birds can form sound–shape associations [23]. The Bayesian coupling‐prior framework [29] provides a principled account of such findings, proposing that pitch–size mappings may emerge from the statistical co‐occurrence of lower frequencies with larger objects and larger animals in the natural environment, even without explicit training [53].

For African penguins, such regularities are particularly relevant in social contexts. Across life stages, vocal parameters encode body‐size‐related information: in chicks, fundamental frequency covaries negatively with body mass and age [33], while in adults of African penguins’ closely related species, both vocalization duration and fundamental frequency reliably signal body size and mass [34, 54]. African penguins can perceive variations in fundamental frequency and formant frequencies within species‐specific vocalizations [35] and integrate auditory and visual cues during individual recognition [36]. Consequently, the ability to extract and align pitch with visual size may support coherent expectations about the physical characteristics of signalers. In densely populated colonies, where visual access to signalers may be limited or delayed, cross‐modal consistency could facilitate efficient recognition of partners, offspring, and other conspecifics.

The behavioral responses that we observed were consistently aligned with the expected low‐pitch/large‐object and high‐pitch/small‐object pairings. Although approach and gaze data showed the same overall pattern, gaze behavior revealed an asymmetry: penguins exhibited a significant preference for congruent stimuli under low‐pitch conditions, but not under high‐pitch conditions. This asymmetry cannot be attributed to perceptual limitations, as African penguins are sensitive across the frequency range used in the study [41]. Instead, the stronger effect for low‐pitched stimuli may reflect the socioecological salience of lower fundamental frequencies, which signal larger conspecifics and likely carry greater weight in intraspecific interactions such as agonistic encounters or mate choice.

The pitch–size congruent responses observed here extended to novel cube sizes and pitch values not encountered during exposure, indicating that the effect was not limited to memory of specific trained pairings. Whether this reflects learning during the exposure phase, a preexisting predisposition, or lifelong experience with natural correlations between object size and sound pitch in the environment remains unresolved [43, 53, 55]. Even without the experimental exposure phase, lifelong experience of natural correlations between object size and sound pitch could have contributed. Future studies could more directly address the developmental origins of these associations in African penguins by experimentally reversing or violating these associations during exposure, or by testing newly hatched chicks prior to substantial multisensory experience.

Exploring cross‐modal associations in non‐human animals offers valuable insight into the evolutionary origins of multisensory integration and the early adaptations that may have shaped its development in humans. Broadening this research across taxa will help clarify whether such associations stem from convergent evolution, where similar perceptual traits emerge independently under comparable socioecological pressures, or from a shared ancestral mechanism, given the evolutionary divergence of mammals, reptiles, and birds around 300–320 million years ago [56].

## 5 Conclusion

Taken together, our findings provide the first experimental evidence that African penguins systematically associate auditory pitch with visual object size. This ability to align information across sensory modalities highlights a flexible form of multisensory processing that may support efficient perception in complex environments. Whether this pattern reflects preexisting predispositions or emerges through experience remains an open question. Nonetheless, the observed behavior demonstrates that the capacity to form and apply abstract multimodal associations is present in an early‐diverging avian lineage, underscoring the potential importance of these cognitive mechanisms in vertebrate evolution.

## Author Contributions

Francesca Terranova: writing – original draft, visualization, validation, methodology, investigation, formal analysis, data curation, conceptualization. Francesca D'Orazio: writing – original draft, methodology, investigation, formal analysis, data curation. Luigi Baciadonna: writing – original draft, validation, methodology, investigation. Cristina Pilenga: writing – review and editing, supervision, resources. David Reby: writing – review and editing, supervision, conceptualization. Livio Favaro: writing – original draft, supervision, methodology, investigation, funding acquisition, conceptualization.

## Conflicts of Interest

The authors declare no conflicts of interest.

## Supporting information




**Supplementary materials**: nyas70313‐sup‐0001‐SuppMat.docx

**Supplementary video**: nyas70313‐sup‐0002‐video.mp4

**Supplementary video**: nyas70313‐sup‐0003‐video.mp4

## Data Availability

The data utilized in this research article are archived on Zenodo (https://doi.org/10.5281/zenodo.14736533).
