# Supplementary material for: Sound Guides Object Size Choices in African Penguins Through Pitch–Size Association
Source: Ann N Y Acad Sci. 2026 Jun 11;1560(1):e70313. doi: 10.1111/nyas.70313 (PMC13255747; doi:10.1111/nyas.70313)
Supplement: Supplementary file 1 — Supplementary materials: nyas70313‐sup‐0001‐SuppMat.docx [file NYAS-1560-0-s002.docx]

**Electronic Supplementary Material**

**Sound guides object size choices in African Penguins through pitch–size association**

**TABLE S1.** Name, date of hatching, sex (F=female, M=male), and provenance of the penguins housed at the Zoomarine (Italy); * Subjects excluded from the test.

| Name | Sex | Hatching date | Provenience |
| --- | --- | --- | --- |
| Africa | F | 10/12/2012 | Burger Zoo, NL |
| Akira | M | 21/02/2013 | Burger Zoo, NL |
| Bianca | F | 15/11/2012 | Bioparc Sable D'Olonne, FR |
| Nino | M | 16/01/2013 | Bioparc Sable D'Olonne, FR |
| July | F | 10/11/2010 | Burger Zoo, NL |
| Rico* | M | 26/02/2010 | Burger Zoo, NL |
| Yve | F | 04/12/2008 | Burger Zoo, NL |
| Soldato | M | 06/11/2009 | Burger Zoo, NL |
| Fiorella | F | 30/11/2006 | Bioparc Sable D'Olonne, FR |
| Gerry | M | 13/12/2003 | Bioparc Sable D'Olonne, FR |
| Kowalsky | M | 29/10/2009 | Burger Zoo, NL |
| Chicco | M | 01/10/2013 | Bioparc Sable D'Olonne, FR |
| Nemo | M | 04/03/2019 | Zoomarine, IT |
| Obelix | M | 20/01/2020 | Zoomarine, IT |
| Alfred | M | 14/04/2020 | Zoomarine, IT |
| Primo | M | 09/01/2021 | Zoomarine, IT |
| Winter | F | 26/11/2021 | Zoomarine, IT |
| Romolo | M | 18/03/2021 | Zoomarine, IT |
| Skipper | M | 25/10/2016 | Zoomarine, IT |
| Rosmarino | M | 21/03/2021 | Zoomarine, IT |
| Dot* | F | 17/02/2023 | Zoomarine, IT |

**TABLE S2.**

Mean and standard deviation of the preferential gaze index for each penguin. Subjects with only one valid observation (e.g., Alfred) have “NA” for the standard deviation.

| Subject | Mean preferential gaze | Standard Deviation |
| --- | --- | --- |
| Africa | 0.0432 | 0.278 |
| Akira | 0.0366 | 0.32 |
| Alfred | 0.308 | NA |
| Bianca | -0.115 | 0.293 |
| Chicco | 0.249 | 0.15 |
| Fiorella | 0.176 | 0.0981 |
| Gerry | 0.454 | 0.328 |
| July | 0.164 | 0.0959 |
| Kowalski | -0.2 | 0.217 |
| Nemo | 0.225 | 0.524 |
| Nino | 0.456 | 0.229 |
| Obelix | 0.0107 | 0.239 |
| Primo | -0.113 | 0.186 |
| Romolo | -0.121 | 0.303 |
| Rosmarino | 0.00427 | 0.217 |
| Skipper | 0.0448 | 0.238 |
| Soldato | 0.133 | 0.367 |
| Winter | 0.184 | 0.254 |
| Yve | 0.386 | 0.203 |

**TABLE S3.**

Mean and standard deviation of the preferential gaze index for each penguin by audio condition.

| Subject | Audio Condition | Mean Preferential Index | Standard Deviation |
| --- | --- | --- | --- |
| Africa | high | -0.104 | 0.324 |
| Africa | low | 0.190 | 0.202 |
| Akira | high | -0.058 | 0.389 |
| Akira | low | 0.226 | NA |
| Alfred | high | 0.308 | NA |
| Bianca | high | 0.069 | 0.255 |
| Bianca | low | -0.300 | 0.236 |
| Chicco | high | 0.249 | 0.150 |
| Fiorella | high | 0.258 | 0.039 |
| Fiorella | low | 0.093 | 0.014 |
| Gerry | high | 0.348 | 0.383 |
| Gerry | low | 0.667 | NA |
| July | high | 0.127 | 0.147 |
| July | low | 0.201 | 0.023 |
| Kowalski | high | -0.307 | 0.306 |
| Kowalski | low | -0.093 | 0.038 |
| Nemo | high | -0.067 | 0.612 |
| Nemo | low | 0.518 | 0.328 |
| Nino | high | 0.573 | 0.151 |
| Nino | low | 0.222 | NA |
| Obelix | high | -0.031 | 0.398 |
| Obelix | low | 0.053 | 0.074 |
| Primo | high | 0.081 | NA |
| Primo | low | -0.210 | 0.112 |
| Romolo | high | -0.270 | 0.224 |
| Romolo | low | 0.176 | NA |
| Rosmarino | high | -0.116 | 0.278 |
| Rosmarino | low | 0.124 | 0.081 |
| Skipper | high | -0.074 | 0.157 |
| Skipper | low | 0.164 | 0.299 |
| Soldato | high | -0.086 | 0.299 |
| Soldato | low | 0.353 | 0.350 |
| Winter | high | 0.237 | 0.214 |
| Winter | low | 0.130 | 0.369 |
| Yve | high | 0.548 | NA |
| Yve | low | 0.304 | 0.207 |

**FIGURE S1.** Stimuli used for the exposure phase with respective sizes s and associated sounds: T3 (23 cm per side; 387 Hz), T1 (22 cm per side 3; 409 Hz), T0 (21 cm per side; 430 Hz), T2 (20 cm per side; 451 Hz), T4 (19 cm per side; 473 Hz).
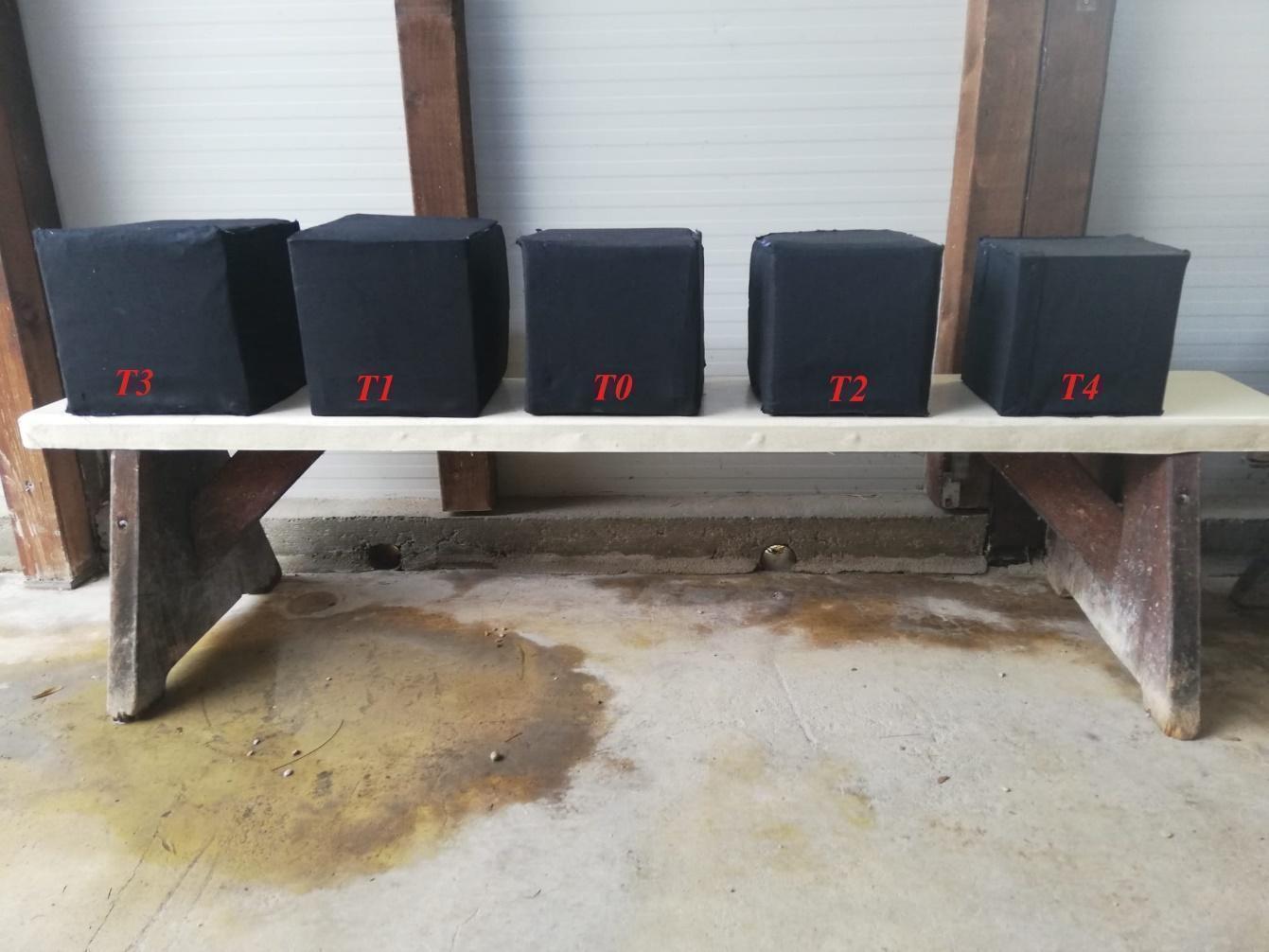


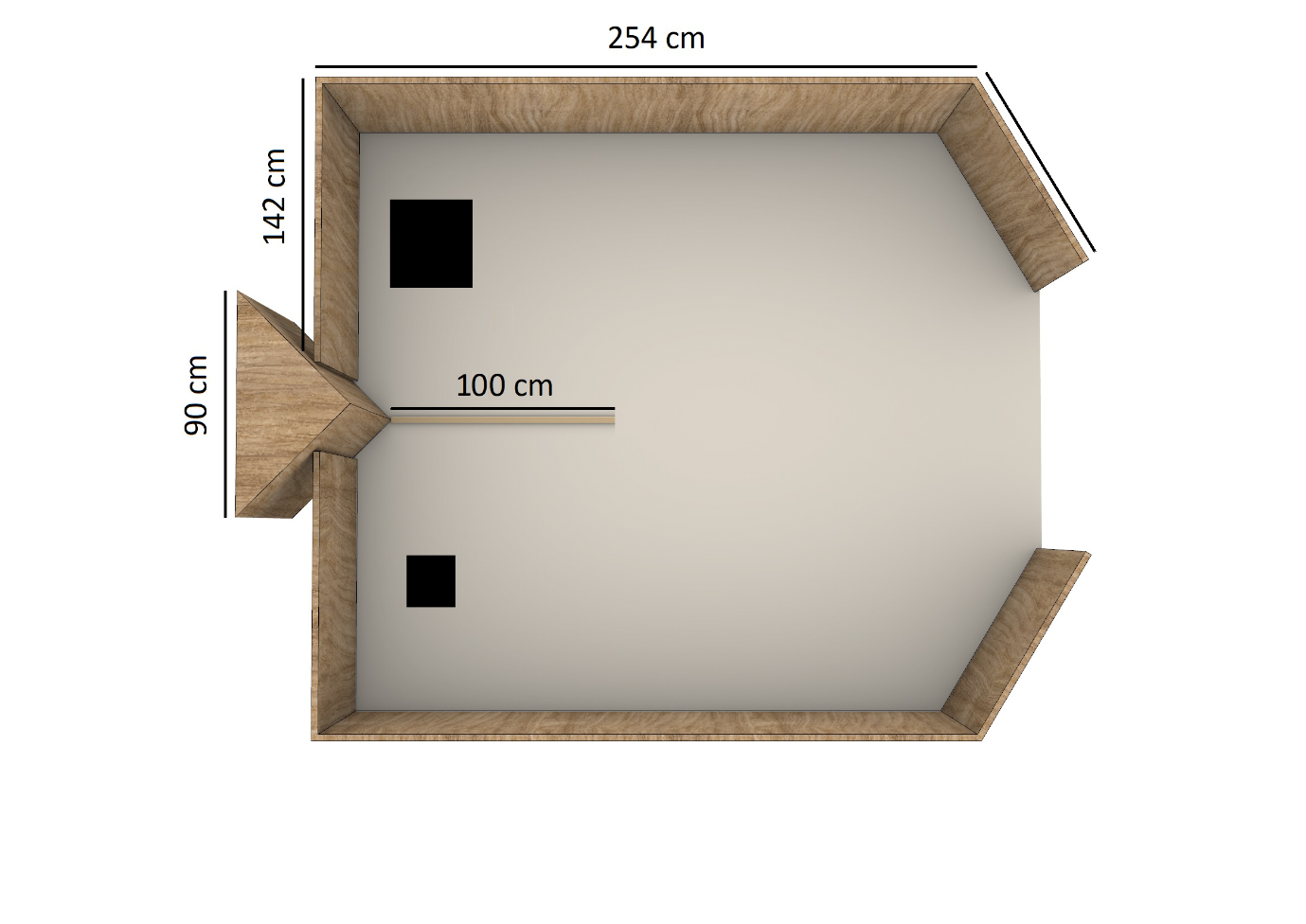


**FIGURE S2.** Setup details of the arena used during the testing phase.


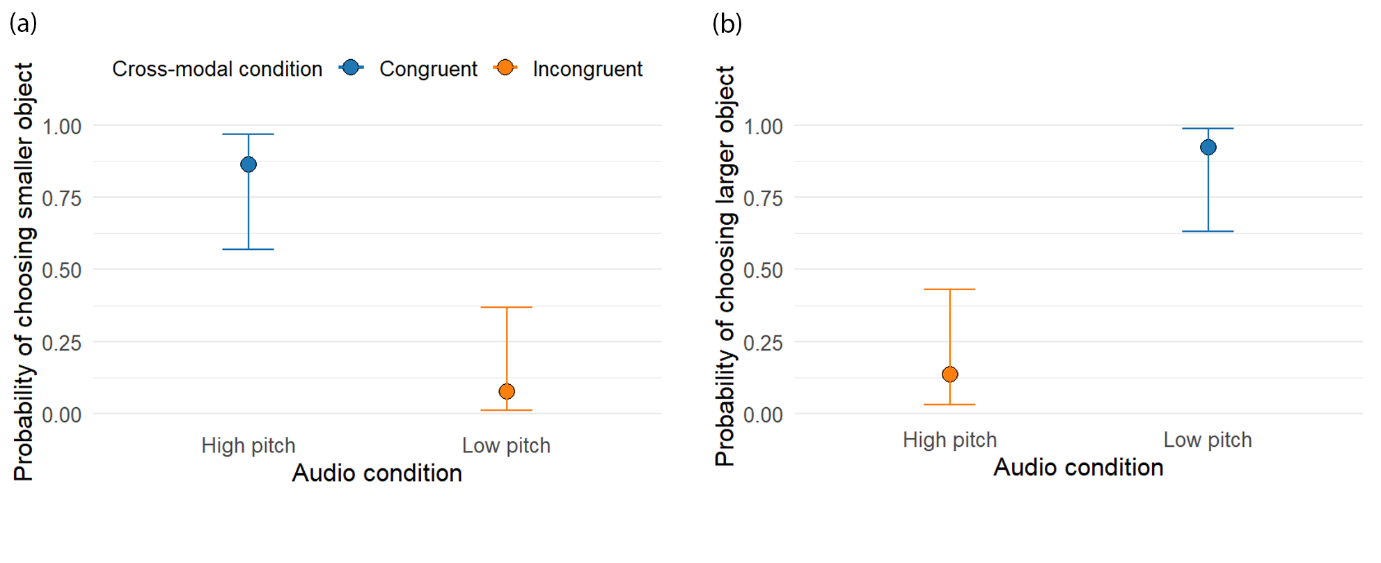
**FIGURE S3.** Effect plots (*ggplot2*) showing predicted probabilities of penguins choosing the smaller (a) or larger (b) object based on high- and low-pitched audio conditions. Plots highlight congruent (blue) and incongruent (orange) cross-modal associations between audio pitch and object size. The vertical error bars indicate 95% confidence intervals around the predicted probabilities.
